# Notes on the biology, distribution, biosecurity status and history in New Zealand of *Macrotrachelia nigronitens* (Stål, 1860) (Heteroptera: Anthocoridae)

**DOI:** 10.3897/BDJ.2.e1077

**Published:** 2014-04-23

**Authors:** Stephen E. Thorpe

**Affiliations:** † School of Biological Sciences (Tamaki Campus), University of Auckland, Auckland, New Zealand

**Keywords:** *Macrotrachelia nigronitens*, New Zealand, Auckland, NZOR, *Anthocoris austropiceus*, Australia, biosecurity, *Gynaikothrips ficorum*, *Teuchothrips disjunctus*

## Abstract

*Macrotrachelia nigronitens* (Stål, 1860) (Heteroptera: Anthocoridae) is permanently present in the wild in Auckland (AK), New Zealand. It should therefore be added to the New Zealand Organisms Register (NZOR). It is a specialised predator of thrips inside leaf-roll galls. It has been present in New Zealand since at least the 1980s. Aspects of its biology, distribution, biosecurity status and history in New Zealand are discussed. The first detailed specimen records from New Zealand are provided, and a biological association is noted for the first time with *Teuchothrips disjunctus* on *Callistemon*, probably its main association in New Zealand, where only two species of thrips cause leaf-roll galls. It has not been found associated with other thrips in New Zealand. *Macrotrachelia nigronitens* is not known to be present in Australia, but the poorly known *Anthocoris austropiceus* Gross, 1954 has been reported, in an easily overlooked publication, to be associated with *Teuchothrips disjunctus* on *Callistemon* in Canberra. This led to an early tentative identification, by the author, of New Zealand material of *Macrotrachelia nigronitens* as *Anthocoris austropiceus*, in collections. This likely misidentification can now be discounted, but further research in Australia is required to determine the true identity of both *Anthocoris austropiceus*, and whatever species of anthocorid was found in Canberra.

## Introduction

**Figure 1.**
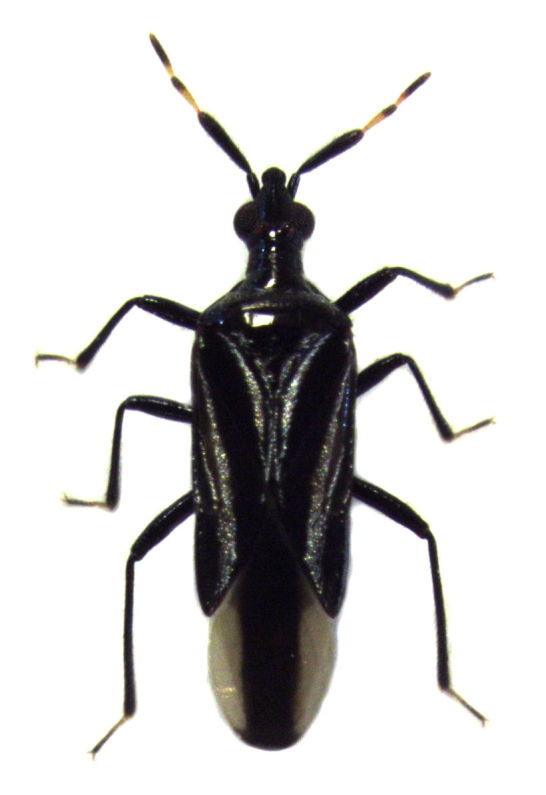
*Macrotrachelia nigronitens* ♂ (length about 3 mm) (AMNZ 87997)

*Macrotrachelia nigronitens* (Stål, 1860) (see Fig. 1) has recently been reported from New Zealand by Larivière and Larochelle (2014), but with few details. They say only (p. 365) that 'The grey literature suggests that this species is present in New Zealand since at least 2006. This can now be confirmed based on comparison between specimens from NZAC and MACN.' No "grey literature" was specified. Such publication of new records without supporting data is not particularly useful. Consequently, the present author herein takes the opportunity to publish data on this species from material currently accessioned in the collections of the Auckland War Memorial Museum (AMNZ), comprising 11 specimens collected between 1997 and 2014. NZAC also holds material of this species but, unfortunately, this material is now unavailable to the author, despite being responsible for most of it. The proper assessment of the biosecurity status of *Macrotrachelia nigronitens* in New Zealand, particularly in terms of how long the species has been present here, requires the tracking down and validation of all relevant material in collections, which has not yet been done, despite all the material being very easily located. Investigations by the author suggest that the species has been present in New Zealand since at least the 1980s. An application (see http://www.epa.govt.nz/Publications/proposal_form_macrotrachelia_spp.pdf) has recently been submitted by Bioforce Limited to the Environmental Protection Authority (EPA) to have *Macrotrachelia nigronitens* declared as "not new", so that it can be legally propagated and/or distributed for the purposes of biological control of pest thrips. In fact, it shall be argued herein that *Macrotrachelia nigronitens* ought never to have been classified as a "new organism" (under the Hazardous Substances and New Organisms (HSNO) Act = not present in New Zealand before 29 July 1998) in the first place, had its biosecurity status been properly assessed. An AMNZ record from 1997 is presented, and an even earlier record from the 1980s is indicated to be held in NZAC. Notes are provided on the biology, distribution and history in New Zealand of *Macrotrachelia nigronitens*.

## Materials and methods

### Institutional abbreviations

AMNZ: Auckland War Memorial Museum, Auckland, New Zealand

AMSA: Australian Museum, Sydney, Australia

MACN: Museo Argentino de Ciencias Naturales “Bernardino Rivadavia”, Buenos Aires, Argentina

NZAC: New Zealand Arthropod Collection, Landcare Research, Auckland

USDA: United States Department of Agriculture

## Taxon treatments

### Macrotrachelia nigronitens

(Stål, 1860)

https://species.wikimedia.org/wiki/Macrotrachelia_nigronitens

Anthocoris nigronitens Stål, 1860: 43 [Original description]

#### Materials

- **Type status:** Other material. **Occurrence:** recordedBy: Keith A. J. Wise; individualCount: 1; sex: female; **Taxon:** scientificName: *Macrotrachelia nigronitens* (Stål, 1860); **Location:** country: New Zealand; stateProvince: Auckland (AK); verbatimLocality: 15 Kingdale Road, Henderson, New Zealand, AK; verbatimElevation: 10 m; decimalLatitude: -36.86036; decimalLongitude: 174.62549; coordinateUncertaintyInMeters: 32; **Identification:** identifiedBy: Stephen E. Thorpe; **Event:** samplingProtocol: Swept; eventDate: 1997-05-09; habitat: Garden; fieldNumber: L5751; **Record Level:** institutionCode: Auckland Museum; collectionCode: AMNZ 59842
- **Type status:** Other material. **Occurrence:** recordedBy: G. Robertson; individualCount: 3; sex: females; **Taxon:** scientificName: *Macrotrachelia nigronitens* (Stål, 1860); **Location:** country: New Zealand; stateProvince: Auckland (AK); verbatimLocality: Auckland Regional Botanic Gardens, Manurewa, New Zealand, AK; verbatimElevation: 40m; decimalLatitude: -37.00879; decimalLongitude: 174.90646; coordinateUncertaintyInMeters: 570; **Identification:** identifiedBy: Stephen E. Thorpe; **Event:** eventDate: 2005-08; habitat: In leaf-roll galls on *Callistemon* sp. infested with *Teuchothrips disjunctus* (Hood); fieldNumber: L14594; **Record Level:** institutionCode: Auckland Museum; collectionCode: AMNZ 73644, 73645, 73646
- **Type status:** Other material. **Occurrence:** recordedBy: John W. Early; individualCount: 5; sex: 3 females, 2 immatures; **Taxon:** scientificName: *Macrotrachelia nigronitens* (Stål, 1860); **Location:** country: New Zealand; stateProvince: Auckland (AK); verbatimLocality: Auckland Airport, Mangere, New Zealand, AK; decimalLatitude: -37.00019; decimalLongitude: 174.79505; coordinateUncertaintyInMeters: 400; **Identification:** identifiedBy: Stephen E. Thorpe; **Event:** eventDate: 2011-04-17; habitat: With *Gynaikothrips ficorum* in rolled leaves of *Ficus microcarpa*; fieldNumber: L19131; **Record Level:** institutionCode: Auckland Museum; collectionCode: AMNZ 85334 (immature), 85335 (immature), 85336 (adult female), 85337 (adult female), 85338 (adult female)
- **Type status:** Other material. **Occurrence:** recordedBy: Stephen E. Thorpe; individualCount: 1; sex: male; **Taxon:** scientificName: *Macrotrachelia nigronitens* (Stål, 1860); **Location:** country: New Zealand; stateProvince: Auckland (AK); verbatimLocality: 25 Felton Mathew Ave, suburb of Saint Johns, New Zealand, AK; verbatimLatitude: -36.87246; verbatimLongitude: 174.84731; coordinateUncertaintyInMeters: 5; **Identification:** identifiedBy: Stephen E. Thorpe; **Event:** eventDate: 2014-03-01; habitat: On *Callistemon* sp. In leaf-roll galls of *Teuchothrips disjunctus*; fieldNumber: L17240; **Record Level:** institutionCode: Auckland Museum; collectionCode: AMNZ 87996
- **Type status:** Other material. **Occurrence:** recordedBy: Stephen E. Thorpe; individualCount: 1; sex: male; **Taxon:** scientificName: *Macrotrachelia nigronitens* (Stål, 1860); **Location:** country: New Zealand; stateProvince: Auckland (AK); verbatimLocality: 2 Farringdon Street, suburb of Glen Innes, New Zealand, AK; verbatimLatitude: -36.87164; verbatimLongitude: 174.85883; coordinateUncertaintyInMeters: 0; **Identification:** identifiedBy: Stephen E. Thorpe; **Event:** eventDate: 2014-03-02; habitat: On *Callistemon* sp., growing over fence into Paddington Reserve. In leaf-roll gall of *Teuchothrips disjunctus*; fieldNumber: EN17241; **Record Level:** institutionCode: Auckland Museum; collectionCode: AMNZ 87997

#### Distribution

Within New Zealand, *Macrotrachelia nigronitens* is present in at least the vicinity of Auckland City and suburbs (AK). The genus *Macrotrachelia* consists of 6 currently recognised Central American species, with only *Macrotrachelia nigronitens* extending (naturally?) into South America (Argentina, Brazil), and possibly also adventive in California (Carpintero 2002, Lewis et al. 2005).

#### Biology

*Macrotrachelia nigronitens* is a predator of thrips inside leaf-roll galls induced by the thrips. Two species of thrips (Thysanoptera: Tubulifera: Phlaeothripidae) are responsible for such galls in New Zealand, both of them adventive. One is *Gynaikothrips ficorum* on *Ficus microcarpa* (Moraceae). The other is *Teuchothrips disjunctus* on *Callistemon* (Myrtaceae). *Ficus microcarpa* is rare in New Zealand, present only in cultivation as an ornamental. *Callistemon* is far more common, and probably represents the main habitat of *Macrotrachelia nigronitens* in New Zealand. Note that *Gynaikothrips ficorum* in New Zealand appears to occur only on *Ficus microcarpa*, and not any of the other, more common species of *Ficus*. Note also that *Macrotrachelia nigronitens* has not been found feeding on any other thrips, at least not in New Zealand.

The data presented herein strongly indicates that *Macrotrachelia nigronitens* is present in New Zealand as a permanent wild population in suburban gardens and parks. It should therefore be added to the New Zealand Organisms Register (NZOR), as exotic and present in the wild.

The pathway of entry of *Macrotrachelia nigronitens* into New Zealand is unknown. It may have arrived here directly from the Americas, associated with *Gynaikothrips ficorum*, and then shifted host to the much more locally abundant *Teuchothrips disjunctus* on *Callistemon*. It is not known to be present in Australia, where both *Teuchothrips disjunctus* and *Callistemon* are native.

## Discussion

In about 2004, the author noticed an unidentified anthocorid specimen in the collection of Auckland Museum (AMNZ 59842), which was collected in the Auckland suburb of Henderson in 1997. Subsequently, Dr. Jocelyn Berry (then hymenopterist at NZAC) gave the author some material collected from Auckland Regional Botanic Gardens in August 2005, and given to her privately by the collector. This material was associated with thrips (*Teuchothrips disjunctus*) in leaf-roll galls on *Callistemon* sp. The species was subsequently found by the author in the same habitat in Auckland Domain. In the then absence of a clue to the identity of the species, the author noticed a published reference by Mound and Walker 1986 to *Anthocoris austropiceus* Gross, 1954. It had been found in leaf-rolls of *Teuchothrips disjunctus* on *Callistemon*, in Canberra, Australia (see p. 83.) The author tentatively identified the New Zealand species as *Anthocoris austropiceus*, based purely on the biological association. The identity of *Anthocoris austropiceus* remains somewhat unclear from the original description in Gross 1954. The Canberra material could not be located, and the (presumably) unique female holotype of *Anthocoris austropiceus* (AMSA K.67861) is apparently mounted on a microscope slide (see http://collections.australianmuseum.net.au/amweb/pages/am/Display.php?irn=1564065), and has not been examined by the author. *Macrotrachelia nigronitens* has not been reported from Australia. Therefore, Australian entomologists are encouraged to double check the true identities of both *Anthocoris austropiceus* Gross, 1954, and whatever species of anthocorid was collected in Canberra (the basis for the identification of the latter as *Anthocoris austropiceus* is unknown, and therefore misidentification cannot at present be ruled out), as it is not impossible that one or other of them could turn out to be *Macrotrachelia nigronitens*. Note that *Anthocoris austropiceus* is the only known native Australian member of the tribe Anthocorini (see http://www.environment.gov.au/biodiversity/abrs/online-resources/fauna/afd/taxa/ANTHOCORINI/checklist), the tribe to which *Macrotrachelia* is also assigned (Carpintero 2002). It therefore appears to be somewhat discordant with the remaining native Australian anthocorid fauna.

On 27 March 2010, the author collected and uploaded to Wikimedia Commons (see https://commons.wikimedia.org/wiki/File:Anthocoris_austropiceus.jpg), as *Anthocoris austropiceus*, an image of a specimen from *Teuchothrips disjunctus* leaf-rolls on a *Callistemon* tree in Auckland Domain. On 31 March 2011, an email was received from David Horton (USDA research entomologist) suggesting that the imaged specimen was in fact a species of *Macrotrachelia*, which he suggested was probably *Macrotrachelia nigronitens*, but with some reservations as the genus has not been taxonomically revised in recent times. Meanwhile, the author noted a specimen in NZAC collected in the 1980s (1986?), by entomologist Dr. Beverley A. Holloway, on a clothes line in her garden at Lynfield, Auckland. The specimen had an identification label on it reading "near *Maoricoris benefactor*", det. M.-C. Larivière. The specimen was with the other material of *Maoricoris* (an unrelated endemic monotypic genus). Full details are currently unavailable to the author, but it is important for this specimen to be tracked down and confirmed as the likely first New Zealand record of *Macrotrachelia nigronitens*. The issue is therefore highlighted herein for others to follow up. Until September 2007, the author deposited several other specimens into NZAC, tentatively identified as *Anthocoris austropiceus*. In 2008 the species was independently discovered in New Zealand (Tamaki Drive, Auckland), associated with *Gynaikothrips ficorum* leaf-rolls on *Ficus microcarpa* (e.g. MPI voucher specimen 09/2008/3333, see http://www.epa.govt.nz/Publications/proposal_form_macrotrachelia_spp.pdf). Lewis et al. 2005 note this as the usual association of *Macrotrachelia nigronitens* in California and elsewhere. The Ministry for Primary Industries (MPI, then MAF) treated this as a new incursion, which resulted in the current biosecurity status of *Macrotrachelia nigronitens* as a new organism in New Zealand. Regrettably, none of the people involved or officially consulted by MPI during the incursion investigation took into account the all of the relevant material in AMNZ and NZAC, material which clearly demonstrates that *Macrotrachelia nigronitens* has been present in N.Z. since at least 1997 and almost certainly at least since the 1980s.

David Horton initially intended to publish the New Zealand record of *Macrotrachelia nigronitens*, but it was apparently subsequently decided, after some correspondence with New Zealand entomologists, that the matter would be best left to them (D. Horton, pers. comm.) Note that the present author had already sent New Zealand material to Horton, who carefully examined it, including dissections of both male and female genitalia, and he determined it as *Macrotrachelia nigronitens*, based on the published literature. Larivière and Larochelle 2014 formally published the identification after 'comparison between specimens from NZAC and MACN', but without giving details how the comparison was made, or upon what the identification of the MACN material is based. Nevertheless, the present author accepts the determination as being certain enough to run with, in lieu of future taxonomic and/or molecular studies.

## Supplementary Material

XML Treatment for Macrotrachelia nigronitens
